# Supplementary material for: Sampling Low Air Pollution Concentrations at a Neighborhood Scale in a Desert U.S. Metropolis with Volatile Weather Patterns
Source: Int J Environ Res Public Health. 2022 Mar 8;19(6):3173. doi: 10.3390/ijerph19063173 (PMC8949442; doi:10.3390/ijerph19063173)
Supplement: Supplementary file 1 [file ijerph-19-03173-s001.zip › TAPS_Supplement_IJERPH.pdf]

Supplemental Materials

Methods

Site Categorization
Regional background sites were not expected to be influenced by specific urban or traffic sources and were located outside urban areas. Urban background sites were expected to be affected by general urban background pollution from vehicle traffic and other sources, but were located in areas with < 3,000 vehicles/day within 50 m. Street sites were expected to be impacted primarily by vehicle traffic and were < 50 m from roads with >10,000 vehicles/day.

Recruiting and Selecting Sites
Once a site resident expressed interest in participating in the study, they were screened via phone or email. To be eligible to participate, the resident had to: 1) have no plans to relocate within the next year; 2) have verbal permission of the site owner or manager to complete sampling; 3) be over 18 years of age; and 4) speak English or Spanish. For the site to be eligible, it had to: 1) be located within the study area of metropolitan Tucson; 2) be free of road construction in the immediate area (within sight or 25 m); and 3) lack plans for construction lasting more than 1 month at the site in the next year. If the participant and site were eligible, we selected the appropriate sampling protocol based on several factors listed in order of importance: availability of 1) a reliable power source; 2) a secure location for air sampling equipment; and 3) unimpeded air flow in the secure space. If the resident confirmed the site evaluation and was interested in the NO_2, NO_x, $PM_{2.5}$, and PM_{10} equipment set up, then we recruited them. If the site and resident failed to meet these criteria, we attempted to recruit them for the NO_2 and NO_x sampling, which was less burdensome.

Next, we scheduled the first of three sampling visits, and collected additional information about site access (e.g., gate/key code, parking restrictions, animals in yard). If a sampling site dropped from the study, they were replaced with another that was of the same site type and sampler set up as geographically close as possible and were sampled for three full visits as was allowable. Site owners were compensated $10 per completed sampling period, up to 3 periods for a total of $30. The University of Arizona Human Subjects Research Review Board determined the project did not constitute human subjects research, but site residents provided written consent.

Site Visits
To avoid atypical air pollution measurements, short periods of time (<2 weeks) that were not representative of normal air pollution concentrations were purposely avoided for sampling, such as major school and work holiday or vacation times. Sites were scheduled as far in advance as possible, with a reminder call or email given seven and two days prior to the planned sampling visit. On at least the initial visit, two study personnel visited the site for safety reasons. After a tour from the participant, the location of sampler setup was determined with their convenience and safety in mind, as well as making sure the space would be available for later sampling visits.

A site survey was completed to determine any potential site-specific or neighboring sources of air pollution that could falsely influence samplers, including pets or livestock, smokers, grills, fire pits, and so on. If these were, the location and number of sources was noted. Also, study personnel requested that the participant and other residents not create any additional sources that could influence the sampler concentrations. If it was not possible to abstain, we

asked participants to keep notes on when and how often the pollution source was active in relation to the sampling visit. The site visit team completed site surveys, noting any item that might influence pollutant measures, such as vegetation, roofs or overhangs, walls, and other structures. GPS coordinates and photos of the set up were also recorded at the sampler location at each visit.

Measuring and Quantifying NO_2 and NO_x

Following ESCAPE, we measured NO_2 and NO_x concentrations with Ogawa Badge Samplers (Ogawa & Company, USA, Inc., Pompano Beach, FL) equipped with weather covers at a height of 2 m (Cyrys et al. 2012) in the same location for each sampling period. NO_2 and NO_x type adsorption filters were always refrigerated and used within one month upon unsealing. The sampler was loaded in the lab right before the sampling trip and then placed in a light-tight container on ice during transport to the site. After the sampler was exposed, it was returned to the light-tight container and put on ice during transport back to the lab, then refrigerated until being analyzed within 24 hours (Ogawa & Co. Inc. 2006; Van Roosbroeck et al. 2006).

All samplers used during a sampling period were analyzed together at the University of Arizona. Briefly, each screen-filter combination was put into a 25 ml glass vial with 8 ml water and shaken immediately. The vial was shaken periodically over the next 30 minutes, cooled to 2-6 °C, and 2 ml of color reagent was added. Next, the sample was shaken quickly and cooled for another 30 minutes. Then, the vial was allowed to equilibrate at room temperature for 20 minutes. Finally, the amount of color derivative was measured with a ThermoScientific Biomate 3 UV spectrophotometer at 545 nm (ThermoScientific, Waltham, MA). While NO_x can be measured to include other oxides of nitrogen (e.g., nitrous oxide (NO_3), for the Ogawa NO_x adsorption filter, NO_x is defined of as the sum of nitric oxide (NO) and NO_2.

Air Sampling Quality Control

As per ESCAPE methods, NO_2, NO_x and PM_{10} field blanks were deployed on 10% of all sampling trips (Eeftens, Tsai, et al. 2012; Cyrys et al. 2012). Ogawa field blanks were taken out of the light-tight container for the time it would take to set up the sampler at the site, and then returned. Similarly, impactors in plastic bags were unsealed during the time it would take to set up the sampling impactors. The limit of detection (LOD) was calculated as three times the standard deviation of the field blanks. Sampler concentrations below the LOD were replaced with the LOD divided by the square root of two (Hornung and Reed 1990). The LOD was assumed to be the same for both $PM_{2.5}$ and PM_{10}, as the same type of filters were used. From each pack of 40 NO_2 and NO_x filters, four were used as laboratory blanks. Duplicates for both size fractions of PM were taken for 10% of site visits. A coefficient of variation for measurement precision was calculated as:

$$CV = \frac{\sqrt{\frac{\sum_{i=1}^{n}(S_i - D_i)^2}{2*n}}}{\frac{\sum_{i=1}^{n}(S_i + D_i)}{2*n}} * 100$$

where *n* is the number of duplicates, *i* is the sampling period, *S* is the sample concentration, and *D* is the duplicate concentration (Cyrys et al. 2012).

Correcting for Temporal Changes

Using ESCAPE methods to account for changes in pollutant concentrations over the year due to meteorology and other factors, we calculated an annual mean concentration for each pollutant at each site as:

$$\bar{x}_{Site} = \frac{\sum_{t=1}^{n} x_{Site,t} - \left(\bar{x}_{Reg} - x_{Reg,t}\right)}{n}$$

where $\bar{x}_{Site}$ is the annual arithmetic mean pollutant concentration at a site; *n* is the total number of measurement periods for a site; $x_{Site,\,t}$ is the concentration at the site during measurement period *t*; $\bar{x}_{Reg}$ is the annual arithmetic mean pollutant concentration at a continuously-running 'background' monitor (unrelated to regional or urban background); and $x_{Reg,\,t}$ is the concentration at the continuously-running background monitor during measurement period *t* (Beelen et al., 2013; Cyrys et al., 2012; Eeftens et al., 2012a). Depending on the change in concentrations over the course of the sampling year and the site's observed concentration during each measurement period, temporal correction could result in negative values.

Results

Quality Control

Field blank concentrations for NO_2 and NO_x were never greater than lab blank values during sample processing. The smallest detected values for site samples for NO_2 and NO_x were 0.16 ppb and 0.88 ppb, respectively. No NO_2 were below the limit of detection (LOD), while on NO_x measure was. The LOD for $PM_{2.5}$ and PM_{10} was 0.77 µg/m^3. Three PM_{10} and one $PM_{2.5}$ measure were below the LOD and were replaced. Coefficients of variation for 8 duplicate samples for NO_2 and NO_x were 5% and 8%, respectively. Coefficients of variation for 8 duplicate samples for $PM_{2.5}$ and PM_{10} were 10% and 12%, respectively.

During PM sampling, none of the pumps ran for <67% of the sampling time or had flow rates less than 8 L/min, however pumps did have diaphragm issues, potentially due to excessive heat exposure. As a result, pumps with developing diaphragm issues could run and be calibrated without issue but eventually became inoperable and were sent in for factory maintenance. Because of this, two urban background sites during different sampling periods in the summer season had no $PM_{2.5}$ or PM_{10} measures for their 3rd of three sampling periods, and another urban background site had no PM_{10} measure for its 2nd sampling period. None of these sites were resampled due to participant disinterest in additional visits. Missing data was not imputed for these failed PM sampling periods. To prevent additional maintenance issues, protection for pumps was developed to reduce heat stress from increased UV radiation (**Figure 2**).

Table S1. Failures to meet ESCAPE quality assurance criteria.

	**n/N (%) Failing to Meet Criteria**			
**Quality Assurance Criteria to Meet**	*NO_2*	*NO_x*	*$PM_{2.5}$*	*PM_{10}*
Measurement $\geq$ LOD	0/112 (0%)	0/112 (0%)	0/54 (0%)	2/52 (4%)
Measurement Total Run Time > 67%	--	--	0/54 (0%)	0/52 (0%)

Calibration Flow Rate > 8 L/min	--	--	0/54 (0%)	0/52 (0%)
